# Assessment of Social Connection among Autistic and Non-Autistic Adults: A Proof of Concept Study for the Connections with Others Scales

**DOI:** 10.3390/healthcare12111077

**Published:** 2024-05-24

**Authors:** Annabelle M. Mournet, Vanessa H. Bal, Edward A. Selby, Evan M. Kleiman

**Affiliations:** 1 Department of Psychology, Rutgers, The State University of New Jersey, New Brunswick, NJ 08854, USA; eas268@psych.rutgers.edu (E.A.S.); evan.kleiman@rutgers.edu (E.M.K.); 2 Graduate School of Applied and Professional Psychology, Rutgers, The State University of New Jersey, New Brunswick, NJ 08854, USA; vanessa.bal@rutgers.edu

**Keywords:** social connection, measure development, neurodiversity

## Abstract

Background: A gap exists in measures available to assess levels of motivation, desire, and value associated with connecting with others. Moreover, few social connection scales have been developed with a goal of including autistic individuals in the sample to create a measure that has utility across neurodiverse populations. This study aims to develop a measure to assess different facets of social connection that is valid among both autistic and non-autistic adults. Methods: The sample consisted of 200 participants recruited online. Participants completed an initial set of 35 items. Exploratory factor analyses and confirmatory factor analyses were performed. Four-factor models were produced by the EFAs. Results: Item reduction resulted in the development of two 8-item scales: the Connections with Others Scale (CWOS) intended for the general population and the CWOS–Autistic Version (CWOS-AV) intended for autistic populations (CWOS-AV). Autistic participants had significantly greater motivation/desire to connect with others compared to non-autistic participants (*t*(195) = 3.39; *p* < 0.001). Conclusions: These measures will allow for greater ability to assess the motivation to connect with others, resulting in improved ability to produce research that clarifies theories and describes psychological phenomena.

## 1. Introduction

Numerous theories have long established connection and relatedness with others to be a basic human need [1,2,3]. “Love and belongingness” is a component of Maslow’s hierarchy of the five essential needs for psychological growth and development [1]. Similarly, Deci and Ryan’s self-determination theory posits that one of the three basic human needs to have consistent volitional motivation is relatedness to others [2]. Most recently, Baumeister and Leary furthered the idea of an inherent motivation to connect with others by suggesting that the desire for interpersonal connections is a “fundamental human motivation” [3].

Beyond existing theories, social connectedness, the perception that one is close and connected to other people [4], is an established protective factor against mental health disorders [5,6]. For instance, social connection has been shown to be an important protective factor for depressive symptoms [7,8], post-traumatic stress disorder symptoms [9], as well as anxiety symptoms [8]. Social connection has also been studied in relation to other psychological phenomena and symptoms, such as interpersonal victimization [10] and suicidal thoughts and behaviors [11,12]. Moreover, one of the leading theories of suicide, the Interpersonal Theory of Suicide [13,14], centers around the impact of poor social connection on suicide risk.

Across studies, loneliness and low levels of social connection are associated with poorer mental health, as has been highlighted in a systematic review by Wang and colleagues [15]. Importantly, the inverse is also consistently true: increased levels of social connection and less loneliness is associated with better mental health and reduced risk for diagnoses [16,17]. There is an increased push for research studies to focus on the role of protective and resiliency factors as features to promote mental health, rather than focusing solely on risk factors [18,19,20]. Consequently, it is important to have a comprehensive ability to assess aspects of well-established protective factors, such as social connection.

### 1.1. Social Motivation Theory of Autism

The role of increasing social connection to support mental health may be particularly relevant among autistic individuals, considering that differences in social communication skills and developing and maintaining relationships are considered core features of autism spectrum disorder. When discussing social connection in autism, it is necessary to acknowledge the social motivation theory of autism [21]. This theory posits that autism represents an “extreme case of diminished social motivation and, as such, provides a powerful model to understand humans’ intrinsic drive to seek acceptance and avoid rejection” [21] (p. 231). This theory stands in stark contrast to the earlier work of Maslow [1], Deci and Ryan [2], as well as Baumeister and Leary [3], all of whom suggested that humans, in general, have an intrinsic motivation and need to connect with others. There has been widespread criticism that this theory fails to acknowledge that many autistic individuals may be highly motivated to connect yet often face social rejection [22]. When discussing the toll of social isolation during the pandemic, den Houting highlights how such misconceptions about autistic people’s motivations and preferences are stigmatizing and overlook barriers to pursuing their desire for social connection [23]. Studies have also provided data that directly contradict the social motivation theory. For example, investigation into objective connections with others has also shown that autistic adults had similar amounts of social groups to non-autistic adults and that most autistic adults (75.5%) report feelings of social identification with at least one group [24]. In other words, autistic adults cannot be assumed to have fewer connections or a reduced desire to have social connections. Building upon the research and voices of autistic individuals, in order to address barriers to social participation, research is needed to advance understanding of the variability and factors affecting autistic adults’ motivation to socially connect. Given this, in the present study, we will compare the factor structure between autistic and non-autistic individuals to explore whether the basic nature (i.e., factor structure) of this construct differs between these groups.

### 1.2. Measurement of Social Connection

The constructs of social motivation and social connection are distinct yet related constructs. To paint a clearer picture of social connection among both autistic and non-autistic individuals, there is a need to consider the concordance between social motivation, perceived connection with others, and tangible connection with others. In doing so, potential discrepancies in desired levels of social connection and existing connections (e.g., high levels of thwarted belongingness or the unmet desire to belong yet very few social connections) may highlight that targeting discrepancies between social motivation and social connection is a useful protective factor against mental health symptoms across individuals regardless of autism status.

While research is needed to investigate the impact of social motivation, it is essential that we do not ignore the distinction between pleasure derived from social interactions compared to an explicit desire and motivation to connect. This issue is particularly clear in the study on the social motivation theory of autism wherein researchers “directly measure social motivation by looking at responses to a questionnaire assessing self-reported pleasure in social and non social situations” [25] (p. 1504). It is not entirely clear, however, that self-reported pleasure in social situations equates to social motivation. There remains a gap in research that takes the further step of assessing pleasure to connect, along with motivation, desire, and the value associated with social connection.

In part, this gap reflects the limited measures available to assess levels of motivation, desire, and value associated with connecting with others. Moreover, few social connection scales have been developed with a goal of including autistic individuals in the sample to create a measure that has utility across neurodiverse populations (i.e., Lubben Social Network Scale [26], UCLA Loneliness Scale [27], Interpersonal Needs Questionnaire [28]). Therefore, the goal of this study is to develop the Connection with Others Scale (CWOS) to measure if a different set of items are optimal for the autistic compared to non-autistic samples, to assess different facets of social connection that are valid among both autistic and non-autistic adults. This study also seeks to investigate whether different facets of social connection (i.e., motivation, desire, value) measured by the CWOS function as distinct factors or if these concepts cluster closely with one another. Finally, we will describe participants’ levels of social connection and desire to connect with others and compare rates between autistic and non-autistic participants. Such an investigation will advance research on ways to best measure and describe the concept of social connection, furthering the ability of researchers to explore assorted components of social connection as a protective factor in mental health research.

## 2. Materials and Methods

### 2.1. Participants

This study contained 200 individuals, half of whom (50%, 100/200) self-reported an autism diagnosis. Recruitment occurred online from December 2021 to May 2022. Table 1 provides a complete breakdown of demographic information.

### 2.2. Procedures

Participants were recruited online. The Simons Powering Autism Research (SPARK) Research Match platform (*N* = 98) and Reddit (*N* = 2) were used to recruit autistic participants. SPARK is a frequently used and well-respected data repository, and existing research has investigated the validity of this sample with regard to autism diagnosis and have found strong evidence in support of the validity of this database [29]. The NIH-funded ResearchMatch platform (*N* = 82) and Reddit (*N* = 18) were used to recruit non-autistic participants. Eligibility criteria for this study were that participants were 18 years or older, able to understand English so as to complete study measures, and legally independent adults, so as to complete the consent form independently. An electronic consent form, as well as study measures, was completed using Qualtrics for all participants. Compensation was in the form of a USD 10 Amazon gift card. This study was prospectively reviewed and approved by the Rutgers Institutional Review Board.

### 2.3. Measures

#### 2.3.1. Social Interaction Items

Participants completed a battery of self-report measures, including an initial set of 35 items to assess the desire, value, enjoyment, and motivation to connect with others (Table 2). Items were created and revised by a clinical psychology graduate student and a team of clinical psychologists. The process involved first reviewing existing measures related to social connection to identify what already existed and identify gaps [26,27,28]. From there, we reviewed existing literature to identify major components of connection. Finally, clinical experience from working with autistic adults was used to address any gaps in the measure. Prior to completing the survey items, participants were told they were completing a questionnaire that was currently being developed and that they would be asked to complete the items and provide thoughts about the quality of the items. The initial item list contained items starting with one of five phrases (“I am”/“I”, “I value”, “I want”, “I enjoy”, and “I am motivated”). Each of the starting phrases was matched with a set of seven ending phrases describing different forms of social connection (“spend time with other people”, “talk with other people”, “get to know other people”, “part of a group of friends”, “interact with people who have similar interests as me”, “interact with people who are similar to me”, and “interact with people who are different than me”). The items used a consistent 7-response Likert Scale, with values ranging from 1 (Strongly Disagree) to 7 (Strongly Agree).

#### 2.3.2. Feedback Measures

In addition to completing the social interaction items, participants were asked to provide feedback on the measure. In the feedback portion, participants were told that they likely noticed a lot of items were worded the same way and that this was carried out purposefully to determine which items were the best to include in the measure. Feedback questions included asking whether any questions were confusing (“Were any of the questions confusing to you? If yes, which parts?”) and if any questions caused discomfort (“Did any questions make you feel uncomfortable? If yes, which questions?”). Participants were asked to elaborate and were also given a spot to provide any additional information. Opinions on items from all participants, rather than a small group of autistic individuals giving perspectives that may not be generalizable to a larger group, were collected to garner large quantities of feedback on the measure that could be incorporated and accounted for when creating the final measure.

#### 2.3.3. Additional Social Connection Measures

To examine convergent and divergent validity, the Interpersonal Needs Questionnaire–Revised (INQ-R [28]) and the UCLA Loneliness Scale [27] were used. The INQ-R is a 15-item self-report measure asking about feelings of thwarted belongingness and perceived burdensomeness, using a 7-point Likert Scale from 1 (Not at all true for me) to 7 (Very true for me). The UCLA Loneliness Scale is a 20-item self-report measure to assess feelings of loneliness. It uses a 4-point Likert scale from 1 (Never) to 4 (Often).

### 2.4. Data Analysis

#### 2.4.1. Qualitative Feedback Data

First, we analyzed the qualitative data provided by participants about the initial set of items and coded responses into several pre-determined sets of themes based on the items. Using a deductive content analysis approach [30], each qualitative response for the confusion and discomfort items was analyzed for the presence of these two issues. Two-sample proportion z-tests were performed to compare differences in confusion and discomfort with the scale (i.e., two of the themes that emerged) among autistic versus non-autistic participants.

#### 2.4.2. Factor Analysis

**Figure 1:**
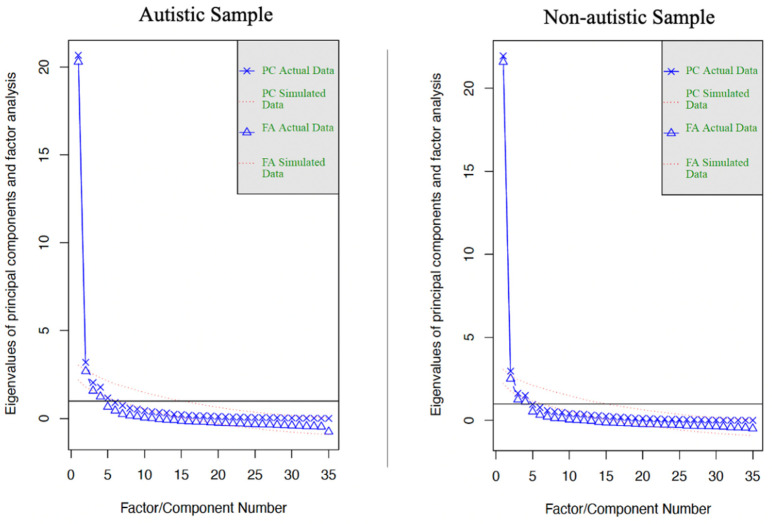
Parallel analysis scree plots.

All quantitative data analysis was performed in R version 4.3.2 (31 October 2023). After analyzing the qualitative data, we conducted a factor analysis with the aim of identifying the factor structure of our initial set of items based on our samples. We randomly divided each sample into two groups, one for the exploratory factor analyses (EFAs) and one for the follow-up preliminary confirmatory factor analysis (CFA) [31]. We utilized four groups: (1) 50 autistic participants for the EFA, (2) 50 non-autistic participants for the EFA, (3) 50 autistic participants for use in CFAs, and (4) 50 non-autistic participants for use in CFAs. We created these groups by using stratified random sampling (i.e., dividing the groups into autistic and not autistic and, then, randomly assigning each group to EFA and CFA). All models were conducted in lavaan [32]. We conducted two EFAs using lavaan, separately for each of the 50-participant groups. We utilized scree plots (Figure 1) to determine the number of factors most appropriate for both EFA groups.

Based on the findings of the EFAs, we performed a series of CFAs. To produce a single measure with acceptable fit for both non-autistic and autistic individuals, we performed a CFA of a model with the items that had the highest factor loadings across both EFAs for each factor, using the remaining 50 autistic and 50 non-autistic participants. Here, we also used measurement invariance analyses to examine the fit for the model utilizing both non-autistic and autistic participants to create one measure. We examined measurement invariance by conducting and comparing four different multi-group SEMs in lavaan [32]. Each model was hierarchical in that it included all the constraints of the prior model plus new constraints. The four models examined were configural, metric, scalar, and strict invariance. Configural invariance tests the equivalency of the factor structures across groups with no constraints applied. Metric invariance examines if factor loadings are invariant across groups. Scalar invariance tests if factor loadings and intercepts are invariant across groups. Strict invariance tests whether residual variances are invariant across groups. Chi-square analyses were used with an aim to compare these models in lavaan using the lavTestLRT function. The chi-squares aimed to compare models with more constraints and higher degrees of freedom to the models with fewer constraints (e.g., configural versus metric, metric versus scalar, and scalar versus strict). Measurement invariance in the comparison is apparent if the chi-square is insignificant. Accordingly, each chi-square was conducted until a significant chi-square test was obtained in order to indicate the specific type of invariance present.

Next, based on the findings of the initial CFA, we computed two additional CFAs using the same two sets of 50 participants. The goal of this CFA was to reduce the number of items on each factor. We selected the two items that had the highest factor loadings for each factor based on the initial CFA for the corresponding group. This decision was made to both ensure that the total number of items was sufficiently reduced while not removing any factors and keeping a consistent number of items across factors. This led to us creating two different models, one for the non-autistic group and one for the autistic group, with overlap in three out of four factors but some differences in the specific items that best loaded on to each factor that were retained in the shorter measure. Finally, to determine whether the model used for the non-autistic CFA would also be acceptable for use among autistic individuals (i.e., to inform future studies where an individual’s autism status is unknown or direct comparability may be desired), we computed a final CFA using the factor structure developed for the non-autistic group while utilizing the autistic sample. Due to the use of two different samples using two different sets of items from the same initial list of items, measurement invariance analyses were not indicated for the second set of CFAs. Acceptable fit of the CFA was based on the fit indices guidelines of Hu and Bentler [33]. A comparative fit index (CFI) of close to 0.95 or greater, a standardized root mean square residual (SRMR) of close to 0.08 or less, and a root mean square error of approximation (RMSEA) of close to 0.06 or less is considered necessary to suggest good fit.

#### 2.4.3. CWOS Outcomes

After the development of the CWOS, a series of independent sample *t*-tests were performed with an aim to compare the total scores on the developed measure among autistic participants compared to non-autistic participants.

#### 2.4.4. Validity Assessment

In line with existing guidelines for scale development and validation, Pearson’s correlations were calculated with an aim to examine convergent and divergent validity [34]. For convergent validity, correlations were examined between the CWOS/CWOS-AV and the INQ-R, given that both measures relate specifically to a desire (though perhaps unmet) for connections with others. For divergent validity, correlations were examined between the CWOS/CWOS-AV and the UCLA Loneliness Scale as these are conceptually both related to social connection but are not expected to be correlated. Correlations were computed separately for the autistic and non-autistic samples.

## 3. Results

### 3.1. Qualitative Feedback

Participants provided qualitative feedback on the initial set of quantitative items. A minority of participants reported discomfort with the items (12.0%; 25/200) and confusion with the items (37.5%; 75/200). There was no significant difference between the proportion of autistic participants that reported discomfort (14%) compared to the proportion of non-autistic participants that reported discomfort (10%; *z* = 0.870; *p* = 0.384). Autistic participants reported significantly more confusion with items (73%; *n* = 73) compared to non-autistic participants (2%, *n* = 2; *z* = 10.370; *p* < 0.001). Based on the high rates of confusion expressed among autistic participants, qualitative responses were analyzed to determine the features that caused confusion.

Out of the 75 participants who reported confusion, the majority of responses that indicated confusion across both samples noted confusion specifically regarding the similar phrasing of many of the items and the seemingly repetitive nature of the items (56%; 42/75). Relatedly, the next most frequently noted reason for confusion among the 75 participants who reported confusion was difficulty understanding the distinction between “motivated”, “value”, and “enjoy” (21.33%; 16/75). Importantly, several other people, including both autistic and non-autistic participants, in the full sample who did not report confusion noted that they liked this feature (5.6%; 7/125), with comments such as “I did like the repeated question methodology of verb substitution to identify actual, practical, and ideal”. Other less frequently endorsed sources of confusion included issues with the lack of ability to provide nuance and context with each answer (8%; 6/75), confusion about who is considered “similar” versus “different” (6.67%; 5/75), and confusion rooted in self-reflection about one’s own experience as opposed to confusion with the questions themselves (2.67%; 2/75). Three participants (4%; 3/75) reported being confused about what was meant by one specific phrase (e.g., confusion about if a “group of friends” refers to “a set of mutual friends that all hang out together or know each other”). One individual did not provide a specific explanation as to what caused confusion (1.33%; 1/75).

Regarding discomfort, the most common reason that individuals expressed discomfort was a result of self-reflection about one’s own experience as opposed to discomfort with the questions themselves (44%; 11/25). For instance, one participant stated: “I felt bad answering that I strongly did not want to interact with other people”. An additional seven individuals reported discomfort with the repetitiveness of the questions (28%; 7/25). One participant expressed discomfort with not being able to provide context with each answer (4%; 1/25). The remaining six participants did not provide specific information about what caused discomfort (24%; 6/25).

### 3.2. EFAs

Scree plots revealed that, for both the autistic and non-autistic EFAs, four factors had an eigenvalue above one (see Figure 1). The Kaiser–Meyer–Olkin criterion (KMO) was 0.926. Visual inspection of the item-factor loadings (Table 2) indicated that the three of the four factors were measuring similar constructs in both the autistic and non-autistic groups but had different items that loaded best on to the factors. All items loaded onto these factors with loadings of 0.50 or higher. These three factors were (1) *valuing getting to know other people (value)*, (2) *interacting with individuals similar to me (similarity)*, and (3) *spending time with other people (time spent).* The fourth factor in the autistic group was focused on *interacting with individuals different from me (difference)*, whereas the fourth factor in the non-autistic group focused on *being part of a group of friends (member)*.

Based on qualitative feedback regarding issues with the repetitive nature of the initial survey list, we retained only the first two items of each factor. In the model utilizing both samples and considering the outcomes of both EFAs, given that there were only three overlapping factors, six items were utilized. The two items selected per factor were those with the highest factor loadings across both EFAs (items 1 [0.82 in non-autistic sample EFA; 0.75 in autistic sample EFA] and 22 [0.76 in non-autistic sample EFA; 0.82 in autistic sample EFA] for the *value* factor, items 6 [0.85 in non-autistic sample EFA; 0.77 in autistic sample EFA] and 8 [0.87 in non-autistic sample EFA; 0.93 in autistic sample EFA] for the *similarity* factor, and items 14 [0.69 in non-autistic sample EFA; 0.62 in autistic sample EFA] and 15 [0.61 in non-autistic sample EFA; 0.54 in autistic sample EFA] for *time spent* factor from the initial list in Table 2).

In the models created based on the separate EFA for each group, both the non-autistic sample model and the autistic sample model contained eight items, with the two items that had the highest factor loading for each of the four factors. The non-autistic sample model was composed of items 1 (0.82) and 7 (0.85) for the *value* factor, items 6 (0.85) and 8 (0.87) for the *similarity* factor, 14 (0.69) and 27 (0.64) for the *time spent* factor, and 17 (0.82) and 29 (0.87) for the *member* factor. The autistic sample model was composed of items 5 (0.89) and 10 (0.87) for the *value* factor, items 6 (0.77) and 8 (0.93) for the *similarity* factor, 2 (0.73) and 14 (0.62) for the *time spent* factor, and 11 (0.84) and 23 (0.85) for the *difference* factor.

### 3.3. CFAs

A CFA was performed on the 6-item 3-factor model using the 50 non-autistic and 50 autistic participants. Through factor invariance testing, this model had scalar invariance (DF = 24; χ^2^ = 19.39; *p* = 0.079); however, the CFI was 0.854, the SRMR was 0.085, and the RMSEA was 0.153 (90% CI = 0.134–0.172; *p* < 0.0001), indicating marginal model fit.

Based on the less-than-optimal fit of the 6-item model utilizing both non-autistic and autistic participants, models of two 8-item measures, one for the non-autistic sample and one for the autistic sample, were tested. CFA of the 8-item 4-factor model for the non-autistic sample (Appendix A) indicated adequate fit (CFI = 0.979, SRMR = 0.044; RMSEA = 0.08, 90% CI = 0.000–0.169; *p* = 0.281). The alpha for this model was 0.89. CFA of the 8-item 4-factor model for the autistic sample (Appendix B) had a CFI of 0.980, an SRMR of 0.064, and a RMSEA of 0.097 (90% CI = 0.000–0.184; *p* = 0.205), indicating adequate fit. The alpha for this model was 0.88. Finally, a CFA using the non-autistic 8-item 4-factor model using the data of the autistic sample, for instances where an individual’s autism status is unknown, had a CFI of 0.958, an SRMR 0.056, and an RMSEA of 0.136 (90% CI = 0.048–0.216; *p*-value = 0.053), indicating suboptimal invariance (Appendix C). The alpha was 0.90 for this model.

Based on these results, the 8-item model derived from the non-autistic sample EFA was used to create the *Connection with Others Scale* (CWOS; Appendix D), and the 8-item model derived from the autistic sample EFA was used to create the *Connection With Others Scale–Autistic Version* (CWOS-AV; Appendix E). Total scores on both the CWOS and CWOS-AV range from 8 to 56. Higher scores denote greater motivation/desire to connect with others. Table 3 contains full information on the properties of both scales, including factor loadings.

### 3.4. CWOS Outcomes

With two confirmed 8-item CWOS scales (CWOS and CWOS-AV; Appendix A and Appendix B), independent sample *t*-tests were performed to compare scores on the CWOS for the autistic versus non-autistic participants. In a comparison of outcome scores, autistic participants had significantly greater scores on the CWOS-AV (mean = 26.48) compared to the scores of non-autistic participants on the CWOS (mean = 20.43) (*t*(196) = 4.55; *p* < 0.001). Additionally, for instances when autism status may not be known, when utilizing the scales designed for each population, autistic participants (mean = 25.28) had significantly greater scores on the non-autistic version of the CWOS compared to non-autistic participants (mean = 20.43) (*t*(195) = 3.39; *p* < 0.001).

### 3.5. Validity Assessment

Regarding convergent validity between the CWOS/CWOS-AV and the INQ-R and UCLA Loneliness Scale, INQ-R scores were significantly correlated both with CWOS scores for non-autistic participants (*r* = 0.45, 95% CI = 0.27, 0.59, *p* < 0.001) and CWOS-AV scores for autistic participants (*r* = 0.41, 95% CI = 0.23, 0.56, *p* < 0.001) with no significant difference between the correlations, based on overlapping 95% confidence intervals. For divergent validity, UCLA Loneliness Scale scores were not significantly correlated with CWOS scores for non-autistic participants (*r* = 0.04, 95% CI = −0.16, 0.23, *p* = 0.723) but were significantly negatively correlated with CWOS-AV scores for autistic participants (*r* = −0.28, 95% CI = −0.46, −0.09, *p* = 0.004). Table 4 provides a complete breakdown of correlation values across social connection measures.

## 4. Discussion

The present study explored the creation of a new measure, the Connection with Others Scale (CWOS). This study represents the first step in the development of the CWOS and CWOS-AV with preliminary evidence supporting the validity of these scales. With detailed feedback from all participants on the scale, next steps will involve an extensive participatory project that works with autistic collaborators in any needed revision of the instrument and works to further test the validity in additional samples.

### 4.1. Psychometric Properties

The factor analyses provided evidence of need for overlapping but distinct constructs comprising social connectedness for autistic and non-autistic adults, thereby providing support for an Autistic Version (CWOS-AV). Further development of the CWOS and CWOS-AV will allow for improved ability to assess constructs related to the motivation and desire to connect with others. Importantly, a considerable strength of the CWOS is the inclusion of a neurodiverse population in the measure’s development, rather than simply using instruments developed for use in non-autistic populations.

Given the less-than-optimal fit of the CFA assessing the 6-item 3-factor model derived from both autistic and non-autistic individuals, we opted to produce two separate measures. The CWOS and the CWOS-AV both had excellent CFI and RMSR. Additionally, the RMSEA values were both acceptable, based on the *p*-value for the RMSEA being greater than 0.05. Importantly, in many instances where the CWOS or CWOS-AV may be used, clinicians and researchers may not be aware of whether an individual has an autism diagnosis, leaving them unsure of the best version to be used. For this reason, we also tested the fit of the standard CWOS among our autism sample and, again, found excellent CFI and RMSR, but suboptimal RMSEA. This suboptimal RMSEA is in line with expectations; if the fit of the standard CWOS for the autistic sample is optimal, this suggests two measures are not needed. Additionally, regarding the adequate but not excellent RMSEAs detected, it is important to consider that RMSEA is a metric that is heavily influenced by degrees of freedom [35], and future studies should therefore seek to replicate the CFA findings of this study using larger samples. Nonetheless, this study provides initial support for the use of the CWOS and CWOS-AV in autistic samples.

In addition to developing scales to assess facets of connections with others, this study also provides a greater understanding of the differences and similarities in numerous aspects of social connection among autistic individuals compared to non-autistic individuals. Of note, differences in the factor structure for the autistic sample versus non-autistic sample were found. These results highlight that when utilizing measures developed based on samples from the general population, “adequate” fit can be detected for autistic samples. However, when we included autistic people in the process of developing the measure, different constructs such as the *difference* factor in the CWOS-AV were identified as being part of the factor structure. Of note, some of the items were loaded on different factors across the CWOS and CWOS-AV versions. It appears that the same item can represent different latent factors in autistic and non-autistic populations. Additionally, whereas correlations with the INQ-R demonstrate that the CWOS/CWOS-AV are significantly associated with another commonly used measure related to desiring social connection, suggesting convergent validity, the lack of correlation between the UCLA Loneliness Scale and CWOS suggests that a construct distinct from loneliness is being captured, establishing divergent validity.

### 4.2. Participatory Research Directions

Autistic participants had significantly greater scores on both versions of the CWOS, demonstrating higher levels of motivation, desire, enjoyment, and value associated with connecting with others. This provides clear evidence in contradiction of the social motivation theory of autism. This, coupled with existing theoretical work [1,2,3] on the basic human need to connect, suggests that the social motivation theory of autism cannot be generalized to all autistic people, or even most autistic people, as is also emphasized by Jaswal and Akhtar [36]. Additional researchers that have critiqued the social motivation theory have specifically suggested engagement in participatory research to better understand and address the needs of autistic individuals in research [37].

In line with the recommendations of Kapp and colleagues [37], we sought to implement the perspectives and thoughts of our participants, both autistic and non-autistic, in the development of this measure. Through analysis of our qualitative data, it was apparent that the majority of negative feedback focused on the repetitive nature of the items, as this caused confusion and even discomfort among participants. Consequently, our data analytic plan focused on substantially reducing the number of items in the measure. We created 8-item measures, resulting in a roughly 77% reduction in the length of the measure. Additionally, by removing a substantial portion of items, we reduced the number of instances in which the ending portion of items were paired with multiple starts (e.g., “I am motivated to”, “I value”, and “I enjoy” all paired with “interact/interacting with people who are different than me”.). This in turn helped to address an additional commonly cited area of confusion by our participants in which differentiating between these starting verbs was difficult. Importantly, by systematically including the perspectives of all participants, we were able to understand that while some participants found difficulty with the approach of creating items by matching different starting verbs with ending actions, this was not universally the case. Some participants specifically noted enjoying this feature as it allowed them to reflect. Consequently, we opted to not fully remove this component of the measure. Future steps related to the use of this tool will involve further participatory research efforts centered around making any needed revisions to the tool and, then, engaging in further testing of the tools.

### 4.3. Research and Clinical Implications

The CWOS and CWOS-AV have utility for use in a variety of areas for future studies. In particular, the CWOS/CWOS-AV may be useful in research related to the Interpersonal Theory of Suicide (IPTS). The IPTS assumes that Baumeister and Leary’s [3] concept of thwarted belongingness is a driver of suicidal thoughts and behaviors (STBs). Importantly, thwarted belongingness would likely only be a driver of STBs if there is in fact a motivation and enjoyment of social connection. Therefore, studies rooted in the IPTS may benefit from examining how facets of connections with others, as assessed via the CWOS/CWOS-AV, impact the relationship between factors such as thwarted belonging and STBs. Enhanced knowledge surrounding a potential mediating variable of motivation or desire to connect with others between thwarted belonging and STBs may increase the ability of researchers to optimize the impact of interventions that target thwarted belonging as a conduit to decreasing suicide risk.

The CWOS/CWOS-AV may also have utility in advancing the understanding of mental-health-related phenomena such as bullying. As in the case of STBs, the extent to which interpersonal victimization impacts a person may be contingent on the degree to which the individual values connection with others. Finally, there is a need to consider the concepts of introversion and extroversion. The CWOS/CWOS-AV may be particularly useful in the study of introversion. It is largely assumed that introverts have low social desire, yet some studies have found that introverts with greater social engagement have increased self-esteem compared to introverts with lower levels of social engagement [38]. While introversion and desire and motivation for connection are clearly linked, they are nonetheless distinct concepts, warranting different measures of each. Studies focused on understanding introversion may benefit from simultaneously investigating motivation and desire for certain levels of engagement and connection with others, using the CWOS/CWOS-AV.

These measures can support bridging the construct of social connection and motivation between autistic and non-autistic populations and can bring forth new understanding of sociality, mental health, and support systems between the autistic and non-autistic. Different factor structures revealed in this study may support the double empathy problem highlighted by Milton [39]. Accordingly, treatment research may seek to establish new ways to enhance social connection interventions for autistic individuals that account for these differences in order to ensure better long-term outcomes for autistic individuals across the lifespan. With this in mind, the CWOS/CWOS-AV may have considerable clinical utility and may be of value to clinicians when developing treatment plans, to understand the extent to which a client is motivated towards and desires social interactions, as well as to assist with monitoring patient outcomes.

### 4.4. Limitations

There are several limitations to note. First, autistic participants were recruited through SPARK, which requires adults to be legally independent and has a higher proportion of females than males, therefore limiting generalization to the broader population of autistic adults. Autistic participants also self-reported autism diagnosis, which may impact the reliability of the results, indicating the need for replication. In addition, both autistic and non-autistic samples were predominantly non-Hispanic/Latinx and White. Power was too limited to do moderation analyses to look at the impact of demographics on CWOS/CWOS-AV scores. Future studies should seek to replicate these findings while employing methods of participant recruitment that specifically focus on increasing the racial and ethnic diversity of the sample. Consequently, the findings of this study may not generalize to other populations of autistic individuals. Finally, while autistic perspectives were solicited from the survey, inclusion of autistic people in the development of the items and/or interviews to more fully understand item responses may have provided insights into other ways that autistic people conceptualize social connection. Future studies will seek to leverage community partners in making any needed revisions to the scales prior to further testing among more diverse samples.

### 4.5. Conclusions

Overall, the CWOS and CWOS-AV were developed in a manner to ensure utility among both non-autistic and autistic individuals. These measures will allow for greater ability to assess numerous facets of social connection, including the motivation to connect with others. Insofar as we are able to better understand a variety of aspects related to social connection, we as researchers will have an improved ability to produce research that clarifies theories and describes psychological-related phenomena, such as STBs and interpersonal victimization.

## Figures and Tables

**Table 1:** Demographic characteristics and analyses.

Demographic Variable	Autistic Sample (N = 100) M (SD)/N (%)	Non-Autistic Sample (N = 100) M (SD)/N (%)	Significant Differences *t* or χ^2^
Age	35.9 (12.6); range = 18–68	41.7 (18.8); range = 18–91	*t* = −2.68, *p*-value = 0.008 *
Gender			χ^2^ = 6.88 *p*-value = 0.032 *
Cisgender woman	51 (51%)	70 (70%)	
Cisgender man	31 (31%)	24 (24%)	
Non-binary	15 (15%)	6 (6%)	
Transgender man	2 (2%)	0 (0%)	
Race			χ^2^ = 3.91 *p*-value = 0.141
White	88 (88%)	76 (76%)	
Black	6 (6%)	16 (16%)	
Asian	4 (4%)	4 (4%)	
American Indian/	1 (1%)	1 (1%)	
Alaskan Native			
Ashkenazi	1 (1%)	0 (0%)	
Native Hawaiian/	0 (0%)	1 (1%)	
Pacific Islander			
Prefer not to disclose	0 (0%)	2 (2%)	
Ethnicity			χ^2^ = 0.35 *p*-value = 0.551
Non-Hispanic/Latinx	93 (93%)	95 (95%)	
Hispanic/Latinx	7 (7%)	5 (5%)	
Age of autism diagnosis	25.46 (16.4) range = 3–63	N/A	N/A

N = sample size; M = mean; SD = standard deviation, *t* = *t*-test, χ^2^ = chi-square; * *p* < 0.05.

**Table 2:** Initial item list and EFA outcomes.

Item #	Item	Autistic Sample Factor	Autistic Sample Factor Loading	Non-Autistic Sample Factor	Non-Autistic Sample Factor Loading	Included in Scale?
1	I value talking with other people	Value	0.75	Value	0.82	CWOS
2	I am part of a group of friends	Time spent	0.73	Time spent	0.48	CWOS-AV
3	I want to talk with other people	Value	0.82	Value	0.73	No
4	I interact with people who are similar to me	Value	0.40	Similarity	0.58	No
5	I enjoy talking with other people	Value	0.89	Value	0.75	CWOS-AV
6	I want to interact with people who are similar to me	Similarity	0.77	Similarity	0.85	CWOS and CWOS-AV
7	I enjoy getting to know other people	Value	0.74	Value	0.85	CWOS
8	I want to interact with people who have similar interests as me	Similarity	0.93	Similarity	0.87	CWOS and CWOS-AV
9	I want to interact with people who are different than me	Difference	0.82	Value	0.53	No
10	I want to get to know other people	Value	0.87	Value	0.60	CWOS-AV
11	I enjoy interacting with people who are different than me	Difference	0.84	Value	0.65	CWOS-AV
12	I am motivated to talk with other people	Value	0.77	Value	0.70	No
13	I value interacting with people who are similar to me	Similarity	0.62	Similarity	0.71	No
14	I spend time with other people	Time spent	0.62	Time spent	0.69	CWOS and CWOS-AV
15	I talk with other people	Time spent	0.54	Time spent	0.61	No
16	I am motivated to interact with people who are different than me	Difference	0.63	Time spent	0.59	No
17	I enjoy being part of a group of friends	Value	0.50	Member	0.82	CWOS
18	I value spending time with other people	Value	0.44	Member	0.48	No
19	I value interacting with people who have similar interests as me	Similarity	0.70	Similarity	0.74	No
20	I am motivated to interact with people who have similar interests as me	Similarity	0.52	Similarity	0.72	No
21	I get to know other people	Difference	0.46	Time spent	0.50	No
22	I want to spend time with other people	Value	0.82	Value	0.76	No
23	I value interacting with people who are different than me	Difference	0.85	Value	0.75	CWOS-AV
24	I interact with people who have similar interests as me	Difference	0.36	Similarity	0.64	No
25	I am motivated to interact with people who are similar to me	Similarity	0.54	Similarity	0.79	No
26	I am motivated to spend time with other people	Value	0.65	Value	0.53	No
27	I interact with people who are different than me	Difference	0.76	Time spent	0.64	CWOS
28	I enjoy spending time with other people	Difference	0.47	Value	0.53	No
29	I value being part of a group of friends	Similarity	0.53	Member	0.87	CWOS
30	I am motivated to get to know other people	Value	0.52	Time spent	0.55	No
31	I enjoy interacting with people who have similar interests as me	Difference	0.56	Similarity	0.68	No
32	I am motivated to be part of a group of friends	Time spent	0.61	Member	0.57	No
33	I enjoy interacting with people who are similar to me	Similarity	0.54	Similarity	0.69	No
34	I want to be part of a group of friends	Similarity	0.35	Member	0.76	No
35	I value getting to know other people	Value	0.62	Similarity	0.54	No

**Table 3:** CWOS and CWOS-AV items and factor loadings.

	CWOS		CWOS-AV	
Factor	Item	Factor Loading	Item	Factor Loading
Value	I value talking with other people	0.82	I enjoy talking with other people	0.89
	I enjoy getting to know other people	0.85	I want to get to know other people	0.87
Similarity	I want to interact with people who are similar to me	0.85	I want to interact with people who are similar to me	0.77
	I want to interact with people who have similar interests as me	0.87	I want to interact with people who have similar interests as me	0.93
Time spent	I spend time with other people	0.69	I am part of a group of friends	0.73
	I interact with people who are different than me	0.64	I spend time with other people	0.62
Member/Difference	I enjoy being part of a group of friends	0.82	I enjoy interacting with people who are different than me	0.84
	I value being part of a group of friends	0.87	I value interacting with people who are different than me	0.85

**Table 4:** Correlations between social connection measures.

Non-Autistic Sample	CWOS *r* (95% CI)	INQ-R *r* (95% CI)	UCLA Loneliness Scale *r* (95% CI)
CWOS	--	0.45 (0.27, 0.59) *	0.04 (−0.16, 0.23)
INQ-R	0.45 (0.27, 0.59) *	--	0.29 (0.10, 0.46) *
UCLA Loneliness Scale	0.04 (−0.16, 0.23)	0.29 (0.10, 0.46) *	--
Autistic Sample	**CWOS-AV**	**INQ-R**	**UCLA Loneliness Scale**
CWOS-AV	--	0.41 (0.23, 0.56) *	−0.28 (−0.46, −0.28) *
INQ-R	0.41 (0.23, 0.56) *	--	0.02 (−0.18, 0.21)
UCLA Loneliness Scale	−0.28 (−0.46, −0.28) *	0.02 (−0.18, 0.21)	--

* Correlation statistically significant at *p* < 0.01.

## Data Availability

The data presented in this study are available on request from the corresponding author.

## Appendix D. Connections with Others Scale (CWOS)

Connection With Others Scale

Please judge how much you agree with each of the following statements.

	7 (Strongly Agree)	6 (Mostly Agree)	5 (Slightly Agree)	4 (Neither Agree nor Disagree)	3 (Slightly Disagree)	2 (Mostly Disagree)	1 (Strongly Disagree)
1. I value talking with other people							
2. I want to interact with people who are similar to me							
3. I enjoy getting to know other people							
4. I want to interact with people who have similar interests as me							
5. I spend time with other people							
6. I enjoy being part of a group of friends							
7. I interact with people who are different than me							
8. I value being part of a group of friends							

Scoring Information: Compute totals by adding up the sum of the following

**Total** (Range = 7–58) =

## Appendix E. Connections with Others Scale–Autistic Version (CWOS-AV)

Connection With Others Scale–Autistic Version

Please judge how much you agree with each of the following statements.

	7 (Strongly Agree)	6 (Mostly Agree)	5 (Slightly Agree)	4 (Neither Agree nor Disagree)	3 (Slightly Disagree)	2 (Mostly Disagree)	1 (Strongly Disagree)
1. I am part of a group of friends							
2. I enjoy talking with other people							
3. I want to interact with people who are similar to me							
4. I want to interact with people who have similar interests as me							
5. I want to get to know other people							
6. I enjoy interacting with people who are different than me							
7. I spend time with other people							
8. I value interacting with people who are different than me							

Scoring Information: Compute totals by adding up the sum of the following

**Total** (Range = 7–58) =
